# Cost-Effectiveness and Budget Impact of Emerging Minimally Invasive Surgical Treatments for Benign Prostatic Hyperplasia

**DOI:** 10.36469/jheor.2021.22256

**Published:** 2021-05-06

**Authors:** Bilal Chughtai, Sirikan Rojanasarot, Kurt Neeser, Dmitry Gultyaev, Stacey L. Amorosi, Neal D. Shore

**Affiliations:** 1 Weill Cornell Medicine, New York, NY, USA; 2 Boston Scientific, Marlborough, MA, USA; 3 Certara Germany GmbH, Loerrach, BW, Germany; 4 Carolina Urologic Research Center, Myrtle Beach, SC, USA

**Keywords:** benign prostatichyperplasia, water vapor thermal therapy, prostatic urethral lift, budget impact analysis, minimally invasive surgical treatments, cost-effectiveness analysis

## Abstract

**Background:** Benign prostatic hyperplasia (BPH) is one of the most prevalent and costly chronic conditions among middle-aged and elderly men. Prostatic urethral lift (PUL) and convective water vapor thermal therapy (WVTT) are emerging minimally invasive surgical treatments as an alternative to traditional treatment options for men with moderate-to-severe BPH. This study evaluated the cost-effectiveness and budget impact of PUL and WVTT for men with BPH using long-term clinical outcomes.

**Methods:** The cost-effectiveness and budget impact models were developed from a US Medicare perspective over a 4-year time horizon. The models were populated with males with a mean age of 63 and an average International Prostate Symptom Score (IPSS) of 22. Clinical inputs were extracted from the LIFT and Rezum II randomized controlled trials at 4 years. Utility values were assigned using IPSS and BPH severity levels. Procedural, adverse event, retreatment, follow-up, and medication costs were based on 2019 Medicare payment rates and Medicare Part D drug spending. One-way and probabilistic sensitivity analyses (PSAs) were performed.

**Results:** At 4 years, PUL was associated with greater retreatment rates (24.6% vs 10.9%), lower quality-adjusted life-years (QALYs) (3.490 vs 3.548) and higher total costs (US7393vsUS2233) compared with WVTT, making WVTT the more effective and less costly treatment strategy. The 70% total cost difference of PUL and WVTT was predominantly driven by higher PUL procedural (US5617vsUS1689) and retreatment (US976vsUS257) costs. The PSA demonstrated that relative to PUL, WVTT yielded higher QALYs and lower costs 99% and 100% of the time, respectively.

**Conclusions:** Compared to PUL, WVTT was a cost-effective and cost-saving treatment of moderate-to-severe BPH. These findings provide evidence for clinicians, payers, and health policy makers to help further define the role of minimally invasive surgical treatments for BPH.

## BACKGROUND

Benign prostatic hyperplasia (BPH) is one of the most prevalent and costly chronic conditions among middle-aged and elderly men.^1^ In the United States, approximately 60% of men aged 60 years and older are diagnosed with BPH,^1^ and in 2013 alone, Medicare was estimated to have spent more than US$1.5 billion on BPH-related office and outpatient services.^2^ BPH leads to lower urinary tract symptoms (LUTS), including voiding and storage problems, which negatively affect patient quality of life (QOL).^3^

Available BPH treatment options differ by their degree of invasiveness, efficacy, adverse event (AE) profiles, and cost consequences. First-line therapy for patients with moderate-to-severe LUTS is often pharmacotherapy.^1^ However, adherence rates are low, with only one-third of men complying with their prescribed pharmacological treatment regimen for longer than 6 months.^4^ Historically, transurethral resection of the prostate (TURP) has been considered the gold standard treatment for BPH; however, men undergoing TURP may develop serious AEs^5^ that can lead to reduced QOL and increased health-care costs.^6^ Additionally, the utilization rate of TURP has been steadily declining in the United States and other countries, reflecting a shift to less invasive surgical treatments for BPH.^7,8^

In recent years, less invasive surgical treatments have evolved for patients with BPH. Prostatic urethral lift (PUL; UroLift System, Teleflex, Pleasanton, CA) and convective water vapor thermal therapy (WVTT; Rezūm System, Boston Scientific, Marlborough, MA) are emerging FDA-cleared minimally invasive surgical treatment options for men with moderate-to-severe LUTS due to BPH. For a PUL procedure, permanent intraprostatic implants are inserted between the prostate lobes to relieve prostatic obstruction.^9^ In contrast, WVTT uses radiofrequency to generate water vapor that penetrates prostate tissue interstices and disrupts tissue cell membranes, resulting in necrosis.^10^ Both PUL and WVTT are associated with similar improvements in International Prostate Symptom Scores (IPSS)^11,12^ and LUTS-related AEs tend to be mild to moderate and resolve shortly after the initial procedure.^9,10^

## OBJECTIVES

While the safety and efficacy of PUL and WVTT have been demonstrated,^9–12^ the medium-term economic outcomes of these technologies have not been well-studied, as previous research only assessed the cost-effectiveness of BPH treatment options in the short term.^13^ Further, although PUL and WVTT are two minimally invasive surgical treatments recommended by the American Urological Association Guideline^1^ to treat a majority of men with BPH, they have different safety and treatment durability profiles, thus impacting the associated costs and health outcomes. Therefore, a need exists to examine the cost-effectiveness of PUL and WVTT over a longer time horizon, taking both IPSS and QOL into consideration.^14^ Such evidence could inform coverage decisions and potentially increase patient access to care. The objective of this study was to estimate the cost-effectiveness and budget impact of PUL compared to WVTT for men with moderate-to-severe BPH from a US Medicare perspective.

## METHODS

We conducted a cost-effectiveness analysis (CEA) comparing PUL and WVTT from a US Medicare perspective over a 4-year time horizon. An Excel-based (Microsoft, Redmond, WA) Markov model was developed using safety and efficacy data from the LIFT^9,11^ and Rezum II^10,12^ randomized controlled trials (RCTs). The trials were identified from a previously published systematic review of PUL and WVTT.^15^ To capture short-term changes in IPSS and AEs, a 3-month cycle length was employed in the first year, followed by a 1-year cycle length for years 2 through 4. The 4-year time horizon was selected since BPH is not considered a life-threatening condition, and to avoid extrapolating the clinical data beyond the trial time. The model was populated with a cohort of males with a mean age of 63 and an average IPSS of 22, using the baseline characteristics of patients in the Rezum II trial.^10^ Cost-effectiveness was evaluated using a willingness-to-pay threshold of US$50 000 per quality-adjusted life-year (QALY) gained, a commonly used threshold for CEA research, and presented as an incremental cost-effectiveness ratio (ICER) at years 1 and 4.^16^ To better understand the key cost components and impact of different post-operative global payment periods, a separate Excel-based budget impact model (BIM) was developed to compare average per-patient costs of PUL and WVTT using the same model patient flow and clinical and cost inputs as in the CEA.

### Model Structure and Patient Pathway

**Figure 1.**
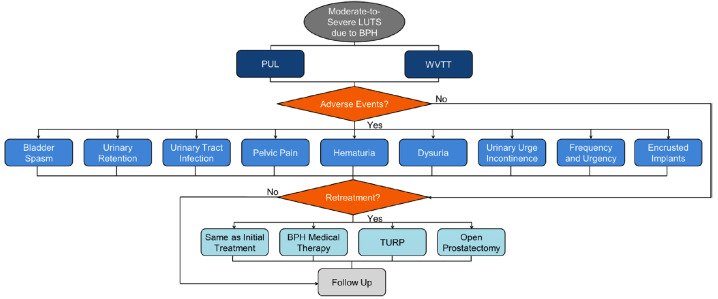
Model Schematic Describing PUL and WVTT Patient Pathway Abbreviations: BPH, benign prostatic hyperplasia; LUTS, lower urinary tract symptoms; PUL, prostatic urethral lift; TURP, transurethral resection of the prostate; WVTT, water vapor thermal therapy. Model schematic depicts the treatment pathways of men with moderate-to-severe LUTS who were treated with either PUL or WVTT. The patients could experience LUTS-related adverse events, require retreatment, or receive follow-up care.

Patients entering the model were assigned to treatment with either PUL or WVTT (Figure 1). After the initial procedure, the model captured the trial-based proportion of patients who required post-procedure catheterization, which impacted patient QOL and costs. Within each model cycle, patients could either experience LUTS-related AEs, require retreatment ranging from BPH medical therapy, the same minimally invasive surgical treatments, and more invasive surgical procedures, or receive follow-up care while simultaneously accumulating costs and utility weights related to each event.

### Clinical Inputs

Clinical inputs for PUL and WVTT, including IPSS, LUTS-related AEs, and retreatment rates, were derived from the LIFT^9,11^ and Rezum II^10,12^ RCTs and are presented in Table 1. These two trials were identified from a previously published systematic review of PUL and WVTT by Tallman et al.^15^ The two RCTs had similar study designs^9,10^ and randomized patients 2:1 to either PUL/WVTT or a sham procedure with rigid cystoscopy. Both trials enrolled men 50 years of age or older with symptomatic BPH, an IPSS of 13 or greater, and a prostate volume between 30-80 cc.^9,10^

**Table 1.** Clinical and Utility Inputs

	**PUL**		**WVTT**		**Utility**	**Mean Time to Recovery (Days)**
	**0-3 Months**	**4-12 Months**	**0-3 Months**	**4-12 Months**		
Mean IPSS (SD)*	22.2 (5.5)^9^	11.5 (7.3)^11^	22.0 (4.8)^10^	10.3 (6.7)^12^	0.99 for mild LUTS^17^ 0.90 for moderate LUTS^17^ 0.79 for severe LUTS^17^	NA
**Post-Procedure Catheterization**	51.4%^9^	NA	90.4%^10^	NA	-0.05^18^	0.9 for PUL^9^ 3.4 for WVTT^10^
**Adverse Events**						
Bladder Spasm	3.6%^9^	0.7%^9^	NA	NA	-0.06^19^	30.0^‡‡^
Urinary Retention	0.7%^9^	0.7%^9^	3.7%^10^	0.0%^10^	-0.18^19^	30.7^‡^
Urinary Tract Infection	2.9%^9^	0.0%^9^	3.7%^10^	0.0%^10^	-0.07^19^	13.3^‡^
Pelvic Pain	17.9%^9^	1.4%^9^	2.9%^10^	0.0%^10^	-0.03**	72.5^‡^
Hematuria	25.7%^9^	0.7%^9^	11.8%^10^	0.0%^10^	-0.20^†^	25.9^‡^
Dysuria	34.3%^9^	0.7%^9^	16.9%^10^	0.7%^10^	-0.03^19^	38.2^‡^
Urinary Urge Incontinence	3.6%^9^	0.7%^9^	0.0%^10^	0.0%^10^	-0.20^19^	30.0^‡^
Frequency and Urgency	7.1%^9^	2.1%^9^	5.9%^10^	0.0%^10^	-0.03**	53.2^‡^
Encrusted Implants	7.1%^9^		NA	NA	-0.03^††^	30.0^‡‡^
**Retreatment Type**						
PUL	21.9%^11^		NA		-0.03^††^	30.0^‡‡^
WVTT	NA		15.4%^12^		-0.03^††^	30.0^‡‡^
BPH Medical Therapy	40.6%^11^		53.8%^12^		-0.03^19^	30.0^‡‡^
TURP	37.5%^11^		23.1%^12^		-0.05^19^	30.0^‡‡^
Open Prostatectomy	0.0%^11^		7.7%^12^		-0.16^20^	30.0^‡‡^

Abbreviations: BPH, benign prostatic hyperplasia; IPSS, international prostate symptom score; LUTS, lower urinary tract symptoms; PUL, prostatic urethral lift; TURP, transurethral resection of the prostate; WVTT, water vapor thermal therapy.

*The mean and standard deviation of IPSS were captured at months 0 and 12.

**The disutility values of pelvic pain and frequency and urgency were not reported in Ackerman et al (2000).^19^ The disutility value of dysuria was applied due to the similarity of the symptoms.

^†^The disutility value of hematuria was assumed as -0.26 in a previously published economic analysis,^21^ which was greater than the disutility value of urinary urge incontinence, the highest disutility value in the model. Thus, the disutility value of urinary urge incontinence was assigned to hematuria.

^††^The disutility values of PUL, WVTT, and encrusted implants were not available. Thus, the disutility values of transurethral microwave thermotherapy reported in Ackerman et al (2000)^19^ was applied.

^‡^Unpublished data from the Rezum II trial.

^‡‡^Mean time to recovery assumptions with clinical validation by the expert were applied as the data were not available from the Rezum II trial.

Following the initial PUL or WVTT procedure, patients could experience improvement, stabilization, or progression of their IPSS relative to baseline in each model cycle. Post-procedural catheterization rates were 51.4%^9^ for PUL and 90.4%^10^ for WVTT with a mean length of catheterization of 0.9^9^ and 3.4 days,^10^ respectively. We assumed that 50% of the catheterization patients required an additional office visit for catheter removal.

LUTS-related AEs for PUL and WVTT were extracted at 0-3 and 4-12 months^9,10^ (Table 1). Between 13-48 months, AE rates for WVTT were 0%^12^ and the AE rates for PUL were assigned as 0%, since the percentage subject-months with AEs reported in the LIFT trial^11^ were low but imprecise. Encrusted implants of patients undergoing PUL that were deployed too proximally were considered an AE in the study.^9^ We assumed that patients who experienced an AE needed one office visit and a prescription drug to treat the AE, except in the case of urinary retention, for which two office visits were assumed for placement and removal of a catheter. LUTS-related AE treatments were validated by a medical expert (Table 2). Since no treatment-related deaths were observed in the RCTs,^9–12^ mortality was not taken into account in this study.

**Table 2.** Cost Inputs

	**Costs**	**Codes and Descriptions**
**Treatment**		
PUL	US$5617	CPT 52441, 52442; HCPCS C9740^22^
WVTT	US$1689	CPT 53854^22^
BPH Medical Therapy	US$415	Office visit (CPT 99213)^22^; tamsulosin 40 mg once daily^23,24^
TURP	US$4793	CPT 52601; DRG 714^22^
Open Prostatectomy	US$7511	CPT 55821; DRG 667^22^
**Office Visit**	US$75	CPT 99213^22^
Post-Procedure Catheterization*	US$75	CPT 99213^22^
**Adverse Events**		
Bladder Spasm	US$91	Office visit (CPT 99213)^22^; oxybutynin 5mg once daily for 14 days^23,24^
Urinary Retention	US$150	Two office visits (CPT 99213)^22^; plus 1-week catheterization
Urinary Tract Infection	US$83	Office visit (CPT 99213)^22^; cotrimoxazole 800 mg/160 mg 2 times/day for 14 days^23,24^
Pelvic Pain	US$82	Office visit (CPT 99213)^22^; ibuprofen 400 mg 4 times/day for 14 days^23,24^
Hematuria	US$75	Office visit (CPT 99213)^22^
Dysuria	US$173	Office visit (CPT 99213)^22^; phenazopyridine 200 mg 3 times/day for 10 days^23,24^
Urinary Urge Incontinence	US$91	Office visit (CPT 99213)^22^; oxybutynin 5mg once daily for 14 days^23,24^
Frequency and Urgency	US$91	Office visit (CPT 99213)^22^; oxybutynin 5mg once daily for 14 days^23,24^
Encrusted Implants	US$3201	CPT 52318^22^

Abbreviations: PUL, prostatic urethral lift; TURP, transurethral resection of the prostate; WVTT, water vapor thermal therapy.

All costs are reported in 2019 US dollars. Medication costs were from 2017 Medicare Part D drug spending and inflated to 2019 dollars using CPI data from the US Bureau of Labor Statistics.

CPT® is a registered trademark of the American Medical Association.

*Post-procedure catheterization was also applied to patients undergoing retreatment with PUL or WVTT.

Patients requiring retreatment could undergo the same initial PUL or WVTT treatment, BPH medical therapy, TURP, or open prostatectomy. Retreatment rates were calculated based upon the CONSORT diagrams from the LIFT^11^ and Rezum II^12^ trials using life-table survival analysis. Retreatment rates were applied to all patients lost to follow-up in the PUL and WVTT treatment groups.

Patients were assumed to require two office visits within the first 3 months following the initial procedure. Thereafter, one office visit per year was assigned for patients with mild and moderate LUTS and two office visits per year were assigned for patients with severe LUTS. Additionally, one post-void residual test per year was assigned for patients with moderate and severe LUTS. The assumptions regarding health-care resource use, including the number of office visits, post-void residual tests, and the proportion of patients who needed an office visit for catheter removal, were all reviewed and validated by a medical expert based on the clinical practice in the United States.

### Health State Utilities

Patient QOL was reflected in the model as health utilities and disutilities. Utility values for BPH severity levels and disutility values associated with post-procedure catheterization, LUTS-related AEs, and retreatments, were derived from the published literature^17–20^ (Table 1). At each model cycle, the mean and standard deviation of IPSS were used to estimate the proportion of patients with mild, moderate, or severe BPH and mapped to the corresponding utility values. The utility values were summed over the model time horizon to obtain overall QALYs. The disutility value of post-procedure catheterization was applied for a fixed time period using the mean duration of catheterization from the LIFT^9^ and Rezum II^10^ trials. Utility decrements for LUTS-related AEs were applied over a finite period of time using the mean time to recovery retrieved from the Rezum II trial (Unpublished data). QALYs were discounted at an annual rate of 3%.

### Cost Inputs

The model included all relevant costs to Medicare: procedural costs, costs associated with AEs, retreatment costs, and follow-up care costs (Table 2). PUL and WVTT procedural costs depended on the site of service. We assumed that 73% of initial procedures occurred in an office setting and 27% occurred in an ambulatory surgery center using the site of service distribution observed in the Rezum II trial (unpublished data). The same site of service distribution was applied for PUL to ensure the consistency of procedural cost calculations between the two procedures, as the distribution observed in the LIFT trial was not available. The procedural cost of PUL was calculated using an average number of 4.9 implants.^9^ Procedural costs, including both physician and facility fees, and office visit costs were based on 2019 Medicare reimbursement rates.^22^ The capital costs of PUL and WVTT were not included as Medicare does not reimburse capital systems separately. In the United States, anesthesia costs are included in the procedural costs of PUL and WVTT; therefore, no additional costs of anesthesia were included. Medication costs for LUTS-related AEs and BPH medical therapy were from 2017 Medicare Part D Drug Spending^23^ and inflated to 2019 dollars.^24^ The CEA used an annual discount rate of 3% that applied to both QALYs and costs. Discounting was not applied in the BIM in order to understand the budget impact of PUL and WVTT at each point in time.^25^ All costs are reported in 2019 US dollars as 2019 was the year that WVTT was assigned a unique Category I CPT Code for reimbursement.

For the BIM, differences in post-operative global payment periods had to be taken into consideration. Post-operative global payment periods are a policy determined by Medicare for medical services “to ensure that Medicare Administrative Contractors pay physicians for the same services consistently across all jurisdictions.”^26^ PUL had a 0-day global period, meaning that the costs of AEs and follow-up care associated with PUL were reimbursed separately following the initial procedure, representing additional costs to Medicare. On the other hand, WVTT had a 90-day global period, meaning that the costs of AEs and follow-up care that occurred within 90 days of the initial procedure were included in the procedural cost and not reimbursed separately.

### Sensitivity Analyses

For the CEA, a one-way sensitivity analysis (OWSA) was conducted to assess the impact of individual parameters on the cost-effectiveness results by varying all clinical and cost parameters by ±10%. A probabilistic sensitivity analysis (PSA) was performed based on 1000 randomly drawn simulations of parameter values and a US$50 000/QALY threshold.

For the BIM, an OWSA was conducted to evaluate the impact of parameter uncertainty on budget impact results by varying all clinical and cost inputs by ±10%. Additionally, a scenario analysis was performed to evaluate the budget impact of different post-operative global payment periods.

## RESULTS

### CEA

**Figure 2.**
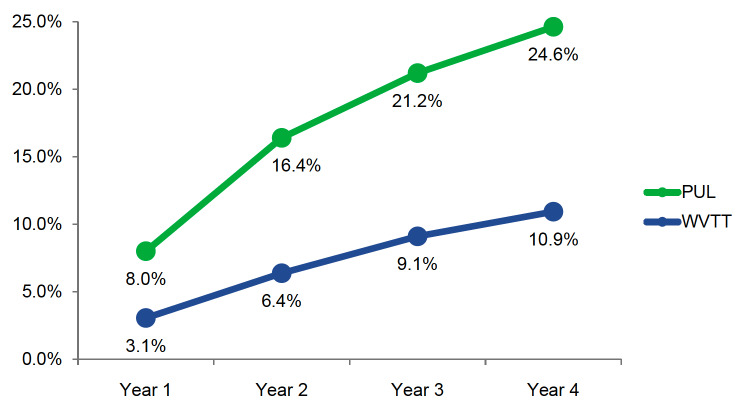
Annual Retreatment Rates of PUL and WVTT from Years 1 to 4 Abbreviations: PUL, Prostatic urethral lift; WVTT, water vapor thermal therapy. Annual retreatment rates of PUL versus WVTT were calculated based upon the CONSORT diagrams from the LIFT^11^ and Rezum II^12^ trials using life-table survival analysis.

PUL was associated with higher retreatment rates compared with WVTT (8.0% vs 3.1%) beginning in year 1 (Figure 2). Retreatment rates at 4 years for PUL and WVTT were 24.6% and 10.9%, respectively.

At 1 year, PUL was associated with lower QALYs (0.917 vs 0.928) and higher total costs (US$6449 vs US$1813) compared to WVTT (Table 3). The same trend continued to year 4, when PUL resulted in lower QALYs (3.490 vs 3.548) and greater total costs (US$7393 vs US$2233) compared with WVTT. Using a willingness-to-pay threshold of US$50 000/QALY, WVTT was a more effective and less costly treatment strategy than PUL for treatment of BPH from years 1 to 4.

**Table 3.** Costs, QALYs, and ICERs at 1 and 4 Years for PUL and WVTT

	**Total Costs**	**Incremental Costs**	**Total QALYs**	**Incremental QALYs**	**ICER vs PUL**
**1 Year**					
PUL	US$6449	–	0.917	–	–
WVTT	US$1813	-US$4636	0.928	0.011	Dominant
**4 Years**					
PUL	US$7393	–	3.490	–	–
WVTT	US$2233	-US$5160	3.548	0.058	Dominant

Abbreviations: ICER, incremental cost-effectiveness ratio; PUL, prostatic urethral lift; QALY, quality-adjusted life-year; WVTT, water vapor thermal therapy.

**Figure 3.**
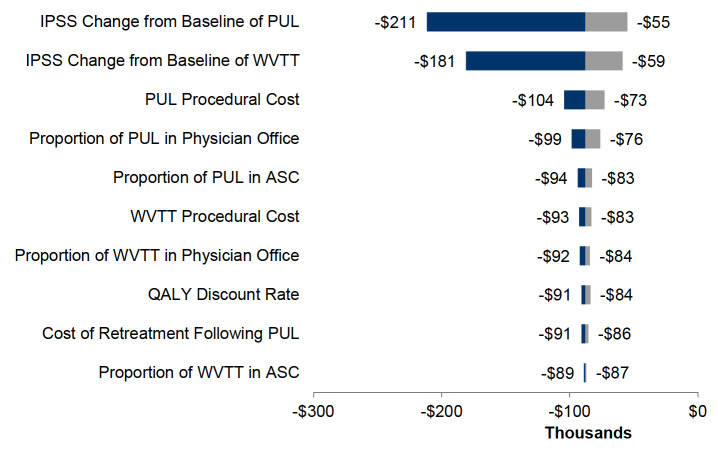
Tornado Diagram of One-way Sensitivity Analysis at 4 Years of PUL Versus WVTT Abbreviations: ASC, ambulatory surgery center; ICER, incremental cost-effectiveness ratio; IPSS, international prostate symptom score; PUL, prostatic urethral lift; QALY, quality-adjusted life-year; WVTT, water vapor thermal therapy. The base case analysis shows an ICER of -US$88 000/QALY at 4 years. The tornado diagram illustrates in which range the ICER varies if the value of the listed model parameter is changed by +10% (dark blue) and -10% (grey), respectively. None of the parameters reaches a positive value (X-axis), which means that under the described conditions, WVTT is always dominant over PUL.

A tornado diagram illustrating the 10 most impactful parameters in descending order of influence on model results at 4 years is depicted in Figure 3. OWSA demonstrated that among all clinical and cost parameters, IPSS change from baseline for PUL and WVTT, and the initial treatment cost of PUL, had the most considerable impact on model results when individually varying each parameter by ±10%.

**Figure 4.**
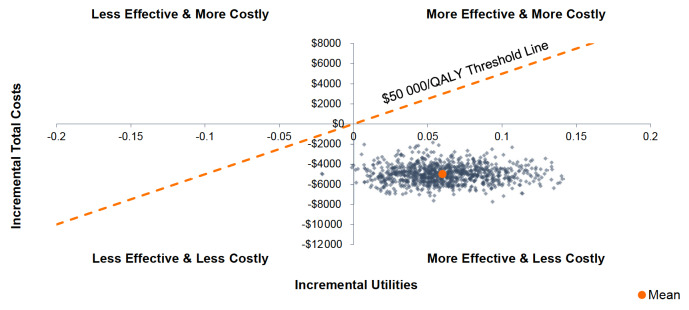
Scatter Plot of Incremental Costs and QALYs Gained at 4 Years for PUL and WVTT Abbreviations: PSA, probabilistic sensitivity analysis; PUL, prostatic urethral lift; QALYs, quality-adjusted life years; WVTT, water vapor thermal therapy. The PSA was derived from 1000 randomly drawn simulations by using varying input values. The grey dots demonstrate the impact on the outcome while the orange dot represents the average of all simulations. Despite the changes in the model values, the majority of the results are below the threshold of US$50 000/QALY.

The PSA simulations (Figure 4) demonstrated that compared with PUL, WVTT led to a lower average total cost of US$4978 (SD, 878) and a greater average QALY of 0.060 (SD, 0.031). As illustrated in the scatterplot, WVTT was less costly than PUL 100% of the time and associated with higher QALYs 99% of the time.

### BIM

**Figure 5.**
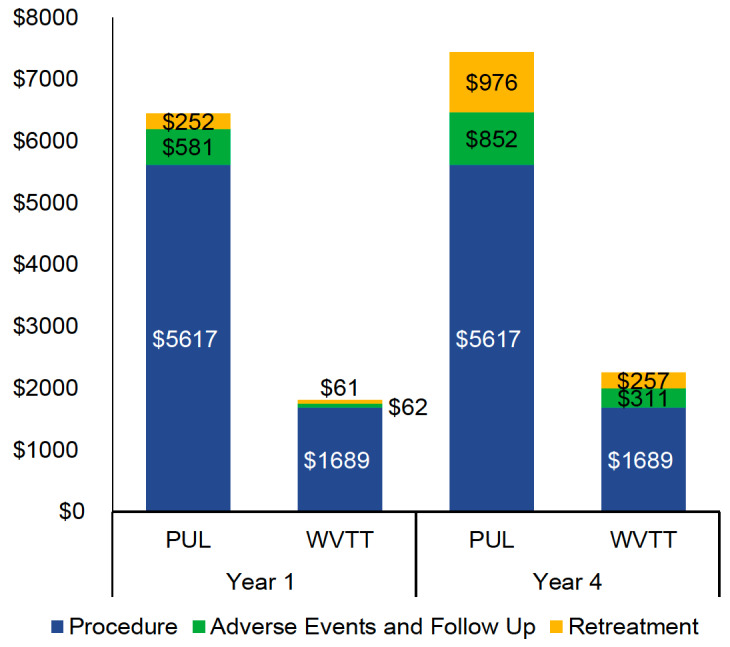
Medicare Per Patient Costs of PUL and WVTT at Year 1 and Year 4 Abbreviations: PUL, prostatic urethral lift; WVTT, water vapor thermal therapy. The stacked bar chart illustrates the cost components for PUL versus WVTT in years 1 and 4. The procedural costs represent the highest cost component for each therapy. WVTT was associated with lower total Medicare costs per patient than PUL at years 1 and 4.

PUL procedural costs were substantially higher than those of WVTT (US$5617 vs US$1689). The results of the BIM demonstrated that PUL was associated with higher total Medicare costs per patient (US$7445 vs US$2257) than WVTT at 4 years (Figure 5). Approximately 70% of the cost difference was attributable to the higher procedural (US$3928) and retreatment costs of PUL (US$719) when compared with WVTT.

When standardizing the global payment periods, PUL costs for AEs and follow-up care at 1 year decreased from US$581 to US$244 with a 90-day global period. The costs of AEs and follow-up care at 1 year for WVTT increased from US$62 to US$302 with a 0-day global period. PUL total costs at 4 years remained substantially higher than WVTT total costs, regardless of the global payment period (US$7445 vs US$2497 with a 0-day global period and US$7099 vs US$2257 with a 90-day global period). OWSA of the BIM demonstrated that WVTT remained cost saving relative to PUL when model parameters were varied by ±10%. PUL and WVTT procedural costs, as well as the costs and rates of retreatment following PUL, were the most impactful model parameters.

## DISCUSSION

This study used long-term efficacy and safety data from the PUL and WVTT RCTs to evaluate the cost-effectiveness and budget impact of PUL and WVTT from a US Medicare perspective for men with BPH experiencing moderate-to-severe LUTS. The CEA showed that WVTT was more effective and less costly than PUL. The BIM demonstrated that WVTT was a cost-saving strategy compared to PUL, primarily due to lower procedural and retreatment costs. Given shared decision-making between health-care providers and men with BPH is at the center of BPH treatment selection,^1^ this study provides economic evidence for key stakeholders to help their patients navigate through different minimally invasive surgical treatment options.

PUL and WVTT are minimally invasive surgical treatment options for the treatment of moderate-to-severe BPH recommended by the American Urological Association Guideline for BPH.^1^ PUL is a mechanical procedure that employs adjustable implants via cystoscopic guidance to elevate the enlarged prostate tissue away from the urethra.^9^ In contrast, WVTT utilizes radiofrequency to create high-pressure water vapor that disrupts prostate gland tissues, resulting in cell death and necrosis.^10^ PUL and WVTT AEs typically occur within 1 year following the initial procedure and are managed in an office-based setting with an oral medication regimen.^9,10^ By year 4, WVTT retreatment rates are less than half of those observed with PUL.

Patients with LUTS due to BPH have been shown to experience a significant reduction in QOL.^27^ This cost-effectiveness analysis differs from previously published research in that our model takes clinical outcomes, costs, and QOL into consideration. This CEA found that WVTT was associated with greater QALYs compared to PUL from year 1 onward. Since neither treatment was associated with mortality, the QALYs represented changes in QOL attributable to the initial procedure, post-procedure catheterization, LUTS-related AEs, and retreatment rates. Although WVTT patients experienced higher post-procedure catheterization rates compared to PUL patients,^9,10^ WVTT resulted in greater QALYs compared to PUL, representing better overall QOL.

Sensitivity analyses demonstrated that the CEA model results were robust to changes in the model parameters. The OWSA revealed that IPSS change from baseline for PUL and WVTT, and the initial treatment costs of PUL, had the greatest impact on model results. The PSA demonstrated that WVTT was less costly than PUL 100% of the time from a Medicare perspective and yielded greater QALYs 99% of the time. Given the robustness of these results, it is highly likely that WVTT would also produce cost savings from other payer perspectives, such as US commercial payers.

In addition to the CEA, the BIM analysis revealed that WVTT had lower per-patient costs compared to PUL, with the key cost drivers being lower procedural and retreatment costs. The procedural cost of WVTT was 3 times less than that of PUL. Additionally, WVTT had lower retreatment rates, thus contributing to lower retreatment costs from year 1 to year 4.

Scenario analyses of the BIM were conducted to understand the impact of standardizing the post-operative global payment periods^26^ for PUL and WVTT, which may have policy implications. Currently, PUL has a 0-day post-operative period, meaning that Medicare reimburses providers separately for AE treatment and follow-up care. In contrast, WVTT has a 90-day post-operative period, so its procedural cost includes AE and follow-up care costs that occur within the first 90 days post-procedure. WVTT remained less costly compared to PUL regardless of the global payment period (ie, 0-90 days). This novel knowledge of the total costs of PUL and WVTT at 4 years after standardizing their post-operative global payment periods may be considered by Medicare when reviewing the payment policy for minimally invasive surgical treatment options for BPH.

This study provides insight into the QOL impact to patients and the long-term costs to Medicare that result from treatment of BPH with PUL and WVTT. To our knowledge, there was only one other published CEA that examined the cost-effectiveness of PUL and WVTT. Ulchaker et al. assessed the cost-effectiveness of PUL, WVTT, conductive radio frequency thermal therapy, pharmacotherapy, photovaporization of the prostate, and TURP, and found that WVTT was more cost-effective compared to PUL.^13^ Despite the consistent conclusion between the two analyses, our study provided several advantages over the previous research. First, our study provided long-term CEA and BIM results using 4-year follow-up data from the LIFT^11^ and Rezum II^12^ trials, as compared to the 2-year follow-up data used in the earlier study. In addition, our study reported QALYs, which incorporates both clinical and QOL outcomes as the effectiveness measure of treatment, whereas IPSS alone was used in the former study. QALY is one single metric for valuing health outcomes that combines both quantity and quality of life^14^ and is often used by health technology associations and health-care decision makers to inform coverage decisions. Since QOL outcomes in this study captured IPSS change from baseline, post-procedure catheterization, LUTS-related AEs, and retreatments for PUL and WVTT the same way, this study demonstrated the cost-effectiveness of PUL compared to WVTT. Lastly, our study accounted for the difference in costs associated with performing PUL or WVTT in different settings of care, while the previous research did not.

There are a few limitations of this study. First, all model-based analyses are subject to result uncertainty and biased input. To minimize these modelling issues, we performed a number of sensitivity analyses and engaged urology and health-care finance experts to validate model assumptions and inputs. Second, our analyses were based on the clinical outcomes of the LIFT^9,11^ and Rezum II^10,12^ trials, as direct comparison studies of PUL and WVTT are not available. The overall trial design and patient inclusion and exclusion criteria for these two trials are similar and the baseline characteristics of the patients treated with PUL or WVTT are not significantly different. However, as the clinical data used in this study were based on the two RCTs, the care pathway may be different than in real-life practice. In addition, this study only evaluated the cost-effectiveness and budget impact of PUL and WVTT from a US Medicare perspective. Different health-care system perspectives in other countries and other BPH surgical procedures, such as TURP, were not studied. Further research is needed to understand the health economic outcomes of PUL and WVTT when adopting these two technologies in other countries and when comparing with other existing procedures.

## CONCLUSIONS

WVTT was the dominant (more effective and less costly) treatment strategy compared to PUL for the minimally invasive treatment of moderate-to-severe LUTS associated with BPH. WVTT was a cost-saving treatment option to Medicare relative to PUL, and the cost difference was predominantly driven by the lower procedural and retreatment costs of WVTT. These findings provide compelling evidence for clinicians, payers, and policy makers to help differentiate minimally invasive surgical treatments for BPH.

### TITLE AND AFFILIATION

Bilal Chughtai, MD, is Associate Professor at the Department of Urology, Weill Cornell Medicine (bic9008@med.cornell.edu); Sirikan Rojanasarot, PhD, is Principal Health Economist at Boston Scientific (sirikan.rojanasarot@bsci.com); Kurt Neeser, DVM, MPH, is Senior Director at Certara Germany GmbH (kurt.neeser@certara.com); Dmitry Gultyaev is Senior Analyst at Certara Germany GmbH (dmitry.gultyaev@certara.com); Stacey L Amorosi, MA, was Director, Health Economics Center of Excellence at Boston Scientific at the time of writing; Neal D Shore, MD, FACS, is Medical Director at Carolina Urologic Research Center (nshore@gsuro.com).

### AUTHOR DISCLOSURE

Drs. Bilal Chughtai and Neal Shore are paid clinical consultants to Boston Scientific. Sirikan Rojanasarot is a full-time employee and stock holder of Boston Scientific. Kurt Neeser and Dmitry Gultyaev are full-time employees at Certara Germany GmbH. Certara Germany GmbH received consultation fees from Boston Scientific for the economic analysis and for the support in writing the manuscript. Stacey Amorosi was a full-time employee of Boston Scientific at the time of writing.
